# Knowledge, attitude, and practices of Filipino adult neurologists on obstructive sleep apnea among stroke patients

**DOI:** 10.1016/j.sleepx.2023.100091

**Published:** 2023-10-15

**Authors:** Pearl Angeli Diamante, Maria Cecilia Jocson, Artemio Roxas

**Affiliations:** a Lung Center of the Philippines, Quezon City, Philippines; b The Medical City, Institute of Neurological Sciences, Pasig City, Philippines

**Keywords:** Knowledge, attitude, and practice (KAP), Neurologists, Obstructive sleep apnea (OSA), Stroke

## Abstract

**Objectives:**

This study aimed to describe the knowledge, attitudes, and practices of Filipino adult neurologists in the recognition and treatment of obstructive sleep apnea (OSA) among patients presenting with acute stroke.

**Methodology:**

A prospective cross-sectional study was conducted using a web-based survey from April to June 2022 among active locally-practicing adult neurology fellows of the Philippine Neurological Association. The 18-item knowledge statements from the validated “Obstructive Sleep Apnea Knowledge and Attitudes (OSAKA) Questionnaire was used as survey instrument. There were also eight additional items assessing knowledge, six items assessing attitudes, and ten items assessing practices that were included.

**Results:**

A total of 119 neurologists participated in the survey. Two-thirds of the respondents were females, and 70 % were between 31 and 40 years old. Majority of the respondents are General Neurologists (57.1 %) followed by Neurophysiologists (10 %) and Stroke Specialists (10 %). Forty-seven percent of neurologists got more than or equal to 75 % of the knowledge statements included in the OSAKA questionnaire correctly. Less than half of the respondents correctly answered the questions on (1) uvulopalatopharyngoplasty as curative for majority of patients with OSA (32.8 %), (2) continuous positive airway pressure (CPAP) therapy can cause nasal congestion (42.9 %), (3) laser-assisted uvuloplasty as treatment for severe OSA (16.8 %), and (4) less than 5 apneas is normal in adults (48.7 %). Majority (>80 %) of the respondents were able to correctly answer the statements relating OSA and stroke. Almost all agreed that OSA as a clinical disorder (95 %) is important and that acute stroke patients with possible OSA needs to be identified (94.1 %) and further evaluated (96.6 %). On the other hand, less than half of the respondents feel confident in: identifying patients at-risk for OSA (47.9 %), ability to manage acute stroke patients with OSA (34.5 %), and ability to manage acute stroke patients with OSA on CPAP therapy (21 %). Most neurologists would sometimes screen OSA among their patients with acute stroke (55.5 %). Most respondents would only educate their patients on OSA sometimes (43.7 %). With regards to the diagnosis (42 %), risk factors (42 %), and treatment options for OSA (40.3 %), most would discuss them with their patients.

**Conclusion:**

Less than half of neurologists were able to get at least 75 % of the knowledge questions. Majority had difficulty with statements pertaining to surgery as cure for OSA, CPAP therapy causing nasal congestion, and OSA severity classification. Almost all has a positive attitude towards the importance of OSA diagnosis and management; however, there is low confidence among them with regards to their practice in identification and handling of these patients.

## Highlights

• Less than half of neurologists were able to get at least 75 % of the knowledge questions.
• Almost all neurologists have a positive attitude towards the importance of OSA diagnosis and management.
• There is low confidence among neurologists with regards to their practice in identification and handling of OSA patients.

## 1 Introduction

Neurologists play an important role in the recognition of possible risk factors in their patients with stroke. In the Philippines, stroke is the second leading cause of death and has a prevalence of 0.9 %, with ischemic strokes comprising the bulk at 70 % [1]. Risk factors need to be identified early on as stroke is a preventable disease. One risk factor commonly overlooked and underdiagnosed is obstructive sleep apnea (OSA).

OSA is noted to be present in approximately 74 % of ischemic stroke patients. Timely recognition and management of OSA improves the clinical outcome of these patients [2]; hence, it warrants a high level of suspicion especially in stroke patients with complaints of snoring and excessive daytime sleepiness.

This study aims to describe the knowledge, attitudes, and practices of Filipino adult neurologists in the recognition and treatment of OSA among patients presenting with acute stroke. Identifying these variables will be able to help neurologists gain insight on their current knowledge, attitude, and practice that may eventually improve patient care. Results will be valuable, especially as there are no published papers yet among neurologists on this topic.

## 2 Methodology

### 2.1 Study design and participants

**Fig. 1:**
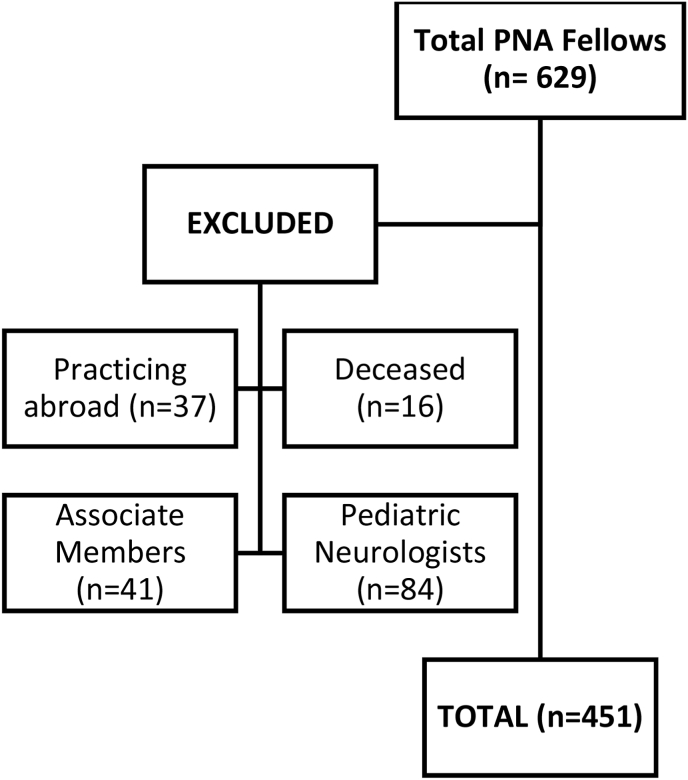
Breakdown of total PNA fellows eligible to participate in the survey.

This was a prospective cross-sectional study among practicing neurologists in the Philippines. Actively practicing adult neurology fellows of the Philippine Neurological Association (PNA), the only recognized organization of Neurologists in the country, were included in the study. As of November 2021, there are a total of 629 fellows in the official roster. Excluded from the roster of eligible participants were pediatric neurologists (n = 84), practicing overseas (n = 37), associate members (n = 41) and deceased (n = 16). (See Fig. 1). Every eligible PNA fellow received both an email and Viber message containing the details of the study, link to the consent form and the survey. To improve the response rate, eligible participants who have not responded after 2 weeks were given another Viber message to encourage participation in the survey.

### 2.2 Study site and period

The study was conducted online through Google Forms which enabled data collection despite social restrictions during the pandemic. Data collection was done between April to June 2022.

### 2.3 Study instrument

The questionnaire was divided into four parts. Part I included the demographic data of participants such as age, gender, location of practice, number of years in practice, residency training institution, sub-specialization (if applicable), and access to sleep laboratory and sleep specialists.

Part II evaluated the knowledge of the participants on OSA. The OSAKA questionnaire (English version), a validated tool to assess the knowledge and attitude of a physician with regards to OSA, was used. Eighteen statements on the symptoms, pathophysiology, epidemiology, diagnosis, and treatment of OSA were provided. Responses were categorized as “true”, “false”, or “do not know”. Each question had a corresponding answer, which was equivalent to one point. The last option “do not know” was scored as an incorrect response. Eight additional statements relating OSA and stroke were added. These statements were based on the expert opinions of the research investigators, backed up by literature and face validated.

Part III assessed the attitude of the participants either by identifying if the statements were “not important”, “somewhat important”, or “important” and “disagree”, “agree”, or “neither agree or disagree”. They were asked for the importance of this condition as a clinical disorder and the importance of identifying the presence of OSA among stroke patients. The six items were patterned from the portion on attitudes in the original OSAKA questionnaire.

Part IV contained questions and statements on the practice of neurologists on availability of resources, screening, and management of OSA. The ten questions/statements were patterned from a previous study by Devaraj last 2020 [3]. The added knowledge statements along with the attitude and practices questions/statements were pilot tested among 5 neurologists, and again, rewording or other modifications were done.

### 2.4 Statistical analysis

Descriptive statistics for the demographic characteristics were presented using mean and standard deviation for quantitative data, and frequency and percentage for qualitative data. Determination of the knowledge, attitudes, and practices of neurologists on OSA among stroke patients were analyzed using frequency and percentage. Sample size was calculated based on the estimation of the population proportion of correct knowledge on OSA among neurologists. Considering that the gold standard of treatment for majority of OSA is continuous positive airway pressure (CPAP) therapy and not uvulopalatopharyngoplasty, OSAKA knowledge question number 2 stating “*Uvulopalatopharyngoplasty is curative for the majority of patients with OSA*” is considered to be one of the most important statements. Assuming that 88.8 % of the respondents got correct answer for question on uvulopalatopharyngoplasty as cure with a maximum allowable error of 5 % and a reliability of 90 %, sample size required is a minimum of 109 [4].

### 2.5 Ethics

This study followed the National Ethical Guidelines for Health and Health Related Research and abided by principles of the Data Privacy Act. It was also approved by the Lung Center of the Philippines – Institutional Ethics and Review Board (LCP-IERB) and the Technical Review Board. All participants were given informed consent forms and were assured of their privacy. Only the researchers and statistician have access to the data gathered and the information will be kept for 2 years.

## 3 Results

Table 1 showed the demographic characteristics of the respondents. There was a total of 119 participants and approximately 2/3 of these were female. Around 70 % of the participants were between 31 and 40 years old, and have been practicing neurologists for 1–10 years. Each of the 19 chapters/clusters of the PNA were represented, except for Region 8- Eastern Visayas and each local current local training institutions have respondents. Majority of the respondents were General Neurologists (57.1 %) followed by Neurophysiologists (10 %) and Stroke Specialists (10 %).

**Table 1:** Demographic data of respondents.

		n (%)
Age	31–40	84 (70.6)
	41–50	21 (17.6)
	51–60	8 (6.7)
	61-above	6 (5)
Gender	Male	40 (33.6)
	Female	79 (66.4)
Location of practice	NCR SE	21 (17.6)
	NCR SW	10 (8.4)
	NCR NE	16 (13.4)
	NCR NW	6 (5)
	CAR	2 (1.7)
	R1	6 (5)
	R2	1 (0.8)
	'R3	11 (9.2)
	R4A	16 (13.4)
	R4B	2 (1.7)
	Marikina	12 (10.1)
	R5	5 (4.2)
	R6	1 (0.8)
	R7	2 (1.7)
	R8	0 (0)
	R9	1 (0.8)
	R10	1 (0.8)
	R11	3 (2.5)
	R12	2 (1.7)
	R13	1 (0.8)
Number of years	Less than 1	25 (21)
practicing	1 to 10	69 (58)
Neurology	11 to 20	14 (11.8)
	21-above	11 (9.2)
Where did you	EAMC	3 (2.5)
have your	JRMMMC	9 (7.6)
residency/	MMC	7 (5.9)
fellowship	PGH	21 (17.6)
training in	QMMC	8 (6.7)
neurology?	TMC	22 (18.5)
	SLMC	14 (11.8)
	UST	16 (13.4)
	UERM	11 (9.2)
	BGH	4 (3.4)
	OTHER	4 (3.4)
Subspecialization	Gen Neurology	68 (57.1)
	Movement	2 (1.7)
	Epilepsy	5 (4.2)
	Neurophysio	10 (8.4)
	Stroke	10 (8.4)
	Dementia	4 (3.4)
	Sleep	2 (1.7)
	Psychiatry	5 (4.2)
	Neurocrit care	3 (2.5)
	Movement/Psychiatry	2 (1.7)
	Epilepsy/Sleep	1 (0.8)
	Neurophysio/Psychiatry	1 (0.8)
	Stroke/Neurocrit care	1 (0.8)
	Others (Headache, Onco, Interventional, Pain, Infectious, Child)	5 (4.2)
	**Total**	**119 (100)**

### 3.1 Knowledge

**Fig. 2:**
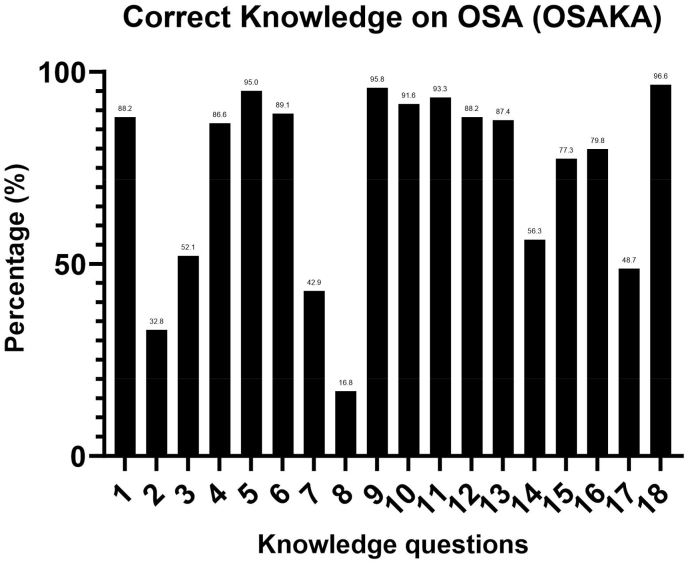
Percentage of correct knowledge on OSA (OSAKA).

Forty-seven percent of neurologists got more than or equal to 75 % of the knowledge statements included in the OSAKA questionnaire correctly. Less than half of the respondents correctly answered the questions on (1) uvulopalatopharygoplasty as curative for majority of patients with OSA (32.8 %), (2) CPAP therapy can cause nasal congestion (42.9 %), (3) laser-assisted uvuloplasty as treatment for severe OSA (16.8 %), and (4) less than 5 apneas is normal in adults (48.7 %). Fig. 2 showed the percentage of correct answers to Knowledge items included in the OSAKA questionnaire.

**Fig. 3:**
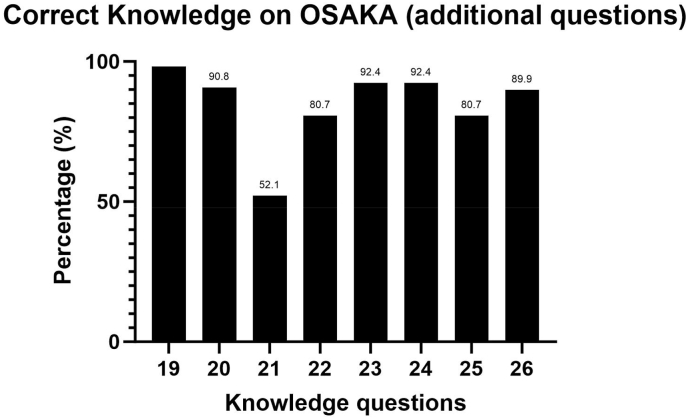
Percentage of Correct Knowledge on OSA (additional items).

Majority (>80 %) of the respondents were able to correctly answer the statements relating OSA and stroke. However, on the statement on OSA as a predictor of good functional outcome after stroke, just over half (52.1 %) of the respondents were able to answer correctly. Fig. 3 shows the percentage of correct answers to Knowledge items relating OSA and stroke.

### 3.2 Attitudes

Almost all agreed that OSA as a clinical disorder, (95 %) was important. Almost all respondents also affirmed that acute stroke patients with possible OSA needed to be identified (94.1 %) and further evaluated (96.6 %). On the other hand, less than half of the respondents felt confident in: identifying patients at-risk for OSA (47.9 %), ability to manage acute stroke patients with OSA (34.5 %), and ability to manage acute stroke patients with OSA on CPAP therapy (21 %). Table 2 summarizes the attitudes of the respondents on OSA and stroke.

**Table 2:** Attitudes of respondents on OSA and stroke.

		n (%)
As a clinical disorder, OSA is	Important	113 (95)
	Somewhat important	6 (5)
	Not important	0 (0)
Identifying patients with possible OSA in acute stroke patients is	Important	112 (94.1)
	Somewhat important	7 (5.9)
	Not important	0 (0)
OSA among stroke patients needs to be further evaluated	Agree	115 (96.6)
	Disagree	0 (0)
	Neither	4 (3.4)
I feel confident identifying patients at-risk for OSA	Agree	57 (47.9)
	Disagree	24 (20.2)
	Neither	38 (31.9)
I am confident in my ability to manage acute stroke patients with OSA	Agree	41 (34.5)
	Disagree	33 (27.7)
	Neither	45 (37.8)
I am confident in my ability to manage acute stroke patients with OSA on CPAP therapy	Agree	25 (21)
	Disagree	51 (42.9)
	Neither	43 (36.1)
	**Total**	**119 (100)**

### 3.3 Practices

A little over half of the responding neurologists would sometimes screen OSA among their patients with acute stroke (55.5 %). Patient characteristics such as being obese, has history of snoring, elderly and stroke in the young were compelling factors that make neurologists screen for OSA.

Less than half were familiar with screening tools such as STOP-BANG (47.9 %) and Epworth Sleepiness Scale (48.7 %), and less than 10% was familiar with Berlin Questionnaire (8.4 %). Among those who were familiar, only about a quarter of the participants would use them all the time (21.4–26.8 %).

More than half of the respondents have access to polysomnography (58.8 %). Majority would request for further evaluation of stroke patients suspected to have OSA (73.1 %) and refer them to a sleep specialist (69.7 %). If they do refer, they do so 2 weeks to 1-month post-stroke (50.5 %).

Most respondents would only educate their patients on OSA sometimes (43.7 %). With regards to the diagnosis (42 %), risk factors (42 %), and treatment options for OSA (40.3 %), most would discuss them with their patients all the time. Half of the respondents attend seminars/lectures on OSA (49.6 %). Table 3, Table 4, Table 5, Table 6, Table 7 summarize the practices of neurologists.

**Table 3:** Screening of OSA among acute stroke patients.

		n (%)
How often do you screen OSA among your patients with acute stroke?	Always	31 (26.1)
	Sometimes	66 (55.5)
	Never	22 (18.5)
	**Total**	**119 (100)**

**Table 4:** Familiarity with OSA screening tools.

		n (%)
Are you familiar with these tools for OSA? [STOP-BANG]	Yes	57 (47.9)
	No	62 (52.1)
Are you familiar with these tools for OSA? [Berlin Q]	Yes	10 (8.4)
	No	109 (91.6)
Are you familiar with these tools for OSA? [Epworth Sleepiness Scale (ESS)]	Yes	58 (48.7)
	No	61 (51.3)
	Total	119 (100)

**Table 5:** Use of OSA screening tools in practice.

		n (%)
Do you use them in your practice? For STOP-BANG	Always	15 (26.8)
	Sometimes	35 (62.5)
	Never	6 (10.7)
Do you use them in your practice? For Berlin Q	Always	2 (22.2)
	Sometimes	6 (66.7)
	Never	1 (11.1)
Do you use them in your practice? For Epworth Sleepiness Scale	Always	12 (21.4)
	Sometimes	33 (58.9)
	Never	11 (19.6)

**Table 6:** Practice on evaluation of OSA patients.

		n (%)
Do you have access to polysomnography?	Yes	70 (58.8)
	No	49 (41.2)
Do you request for further evaluation of stroke patients suspected to have OSA?	Yes	87 (73.1)
	No	32 (26.9)
Do you refer	Yes	83 (69.7)
suspected OSA	No	36 (30.3)
patients to a sleep specialist?		
Do you attend seminars/lectures on OSA?	Yes	59 (49.6)
	No	60 (50.4)
If you do refer your stroke patients who have possible OSA to a sleep specialist, when do you do it?	Immediately	33 (34)
	3–5 days	15 (15.5)
	2 wks-1mo	49 (50.5)

**Table 7:** Practice on patient discussion on OSA.

		n (%)
I educate my patients about OSA	Always	44 (37)
	Sometimes	52 (43.7)
	Never	23 (19.3)
I discuss with my patients on how OSA can be diagnosed (e.g. the need to undergo polysomnography/PSG)	Always	50 (42)
	Sometimes	40 (33.6)
	Never	29 (24.4)
I talk to my patients about OSA risk factors	Always	50 (42)
	Sometimes	44 (37)
	Never	25 (21)
I discuss treatment options for OSA with my patients	Always	48 (40.3)
	Sometimes	42 (35.3)
	Never	29 (24.4)
	**Total**	**119 (100)**

## 4 Discussion

This study represents the first national survey on adult Filipino neurologists assessing their knowledge, attitude, and practices with respect to OSA among acute stroke patients. Other published studies are done internationally which focused on other specialties such as pulmonologists, general physicians, and otorhinolaryngologists. Published local research, the RIFASAF Project, alludes to snoring but does not directly identify OSA as a risk factor for cerebrovascular diseases among Filipinos [5]. However, this study has been an important landmark paper among local physicians and has been evident in the results of our study that snoring somewhat raises concern therefore initiating screening for possible OSA on these stroke patients.

OSA remains to be an underdiagnosed and undertreated condition. While approximately 2–4% of the general population has OSA, it is identified in 73.7 % of ischemic stroke patients. OSA is the most common type of sleep-disordered breathing disorder found after stroke and has been identified as an independent risk factor not just for the development of cardiovascular and cerebrovascular diseases but also increases death [3,[6], [7], [8]]. It can predispose patients to develop other comorbid conditions that also increase the risk for stroke-hypertension, diabetes, obesity, and cardiovascular diseases including atrial fibrillation [3,6]. Adequate knowledge on OSA is therefore important among neurologists because they are usually the ones who manage acute stroke patients.

Due to the importance of the timely recognition and treatment of OSA, Schotland and Jeffe created and validated a questionnaire that assesses physician's knowledge and attitudes about OSA [9]. The OSAKA questionnaire was developed in 2003 and was used since then in multiple published papers. In this study, only 47.1 % of the respondents were able to get at least 75 % of the statements correctly. It was noted that statements concerning surgical procedures serving as treatment for OSA were incorrectly answered, as well as what constitutes a normal value of respiratory events in an adult. This emphasizes the need for bridging gaps in knowledge regarding OSA among neurologists.

In the Philippines, there is minimal exposure to sleep medicine among residency training programs in neurology. As it is a relatively new discipline, the current number of sleep specialists locally is still low and there is no formal set rotation for this subspecialty during residency as of the moment. For some institutions who offer elective programs, it can be taken as a self-funded month-long course overseas. This subject therefore does not get to be discussed often and reinforced during training, hence the poor knowledge among practicing fellows. In the recent past years, sleep and its association to neurological conditions are starting to be recognized as an important part of diagnosis and management among patients. Lectures are becoming more frequent and are incorporated among conferences and conventions.

It is part of the competency of neurologists to identify patients with OSA and be confident in the diagnosis and management of these cases. It is good to note that most of them have a positive attitude on this aspect however, only less than half of the participants are confident in identifying OSA and managing stroke patients with OSA. To aid in identifying the presence of OSA in an individual, screening can be done using questionnaires. However, more than half of the respondents admit to not being familiar with STOP-BANG, Berlin Q, and Epworth Sleepiness Scale, which once again emphasizes the need to improve education among physicians especially neurologists regarding OSA screening, diagnosis and management. A positive attitude towards the importance of OSA diagnosis and management does not always equate into application to practice. It is a start; however, it should not stop there. Confidence in handling OSA patients need practice and proper guidance among experts. Learning on how to diagnose and treat them theoretically might not be enough to put it into practice.

Improving the current situation needs dedication and support from the local sleep medicine society. Continuing education of physicians through lectures and the dissemination of clinical practice guidelines can help increase the knowledge on OSA. Training more sleep subspecialists will also help cater to more Filipinos including those in the far-flung regions of the country.

## 5 Conclusion

This study aimed to increase awareness among Filipino Neurologists regarding their current knowledge, attitude and practices on OSA among stroke patients. Less than half of neurologists were able to get at least 75 % of the knowledge questions. Majority had difficulty with statements pertaining to surgery as cure for OSA, CPAP therapy causing nasal congestion, and OSA severity classification. Almost all has a positive attitude towards the importance of OSA diagnosis and management; however, there is low confidence among them with regards to their practice in identification and handling of these patients.

## 6 Recommendations

The authors would like to recommend more avenues and opportunities that would provide awareness and information on prevalence, burden, diagnosis and management of OSA among neurologists and practicing physicians locally. It is also recommended to initiate epidemiological studies on local population to be able to craft locally applicable healthcare provisions and clinical practice guidelines among Filipinos with OSA. Incorporation of OSA screening tools in current stroke protocol and pathways used in local hospitals may improve identification of OSA suspects and enable appropriately timed management.

## CRediT authorship contribution statement

**Pearl Angeli Diamante:** Conceptualization, Data curation, Formal analysis, Methodology, Writing – original draft. **Maria Cecilia Jocson:** Conceptualization, Methodology, Supervision, Writing – review & editing, of draft. **Artemio Roxas:** Conceptualization, Methodology, Supervision, Writing – review & editing, of draft.

## Declaration of competing interest

The authors declare that they have no known competing financial interests or personal relationships that could have appeared to influence the work reported in this paper.
